# Characterizing sleep-related phenotypes with patient-reported outcomes in Parkinson’s disease

**DOI:** 10.3389/fnagi.2025.1630150

**Published:** 2025-10-16

**Authors:** Sandeep Grover, Celina Chand, Mollie McKenzie Acuff, Joshua Farahnik, Laurie K. Mischley

**Affiliations:** ^1^Institute of Human Genetics, University of Marburg, Marburg, Germany; ^2^Bastyr University Research Institute, Kenmore, WA, United States; ^3^Parkinson Center for Pragmatic Research, Shoreline, WA, United States; ^4^Department of Radiology, University of Washington, Seattle, WA, United States

**Keywords:** insomnia, fatigue, non-motor symptoms, sleep disorders, sleep quality, patient-reported outcomes

## Abstract

**Objective:**

Sleep disturbances are common in Parkinson’s disease (PD) and significantly impact patients’ quality of life. However, the clinical symptoms associated with poor sleep remain underexplored. The Patient-Reported Outcomes in PD (PRO-PD) is a remote patient monitoring tool enabling symptom-level resolution. The goal of this study was to use the PRO-PD to describe symptomatic differences associated with sleep quality.

**Methods:**

A cross-sectional analysis was conducted using baseline data of an ongoing prospective cohort study of patients with idiopathic PD (IPD). Sleep quality was assessed using the Pittsburgh Sleep Quality Index (PSQI), while disease severity was evaluated using the PRO-PD. Multivariable regression analysis was performed to judge the association between PSQI and PRO-PD, adjusting for age, gender, income, and years since diagnosis.

**Results:**

Among 331 participants, 70.2% of patients with IPD reported poor sleep quality (PSQI>5). Furthermore, a significant positive correlation was observed between PSQI scores and PRO-PD scores (*p* = 2.24 × 10^−12^). Multivariable regression analysis confirmed this association, with each unit increase in the PSQI score corresponding to a 39.7-unit increase in the PRO-PD score (*p* = 1.25 × 10^−8^). Sleep quality was most strongly correlated with self-reported insomnia (*ρ* = 0.69), with moderate associations observed for fatigue, anxiety, poor balance, depression, and apathy (*ρ* = 0.29–0.37), and weaker associations with myalgia, visual complaints, cognition, restless legs, gait, posture, and urinary symptoms (*ρ* = 0.20–0.28).

**Conclusion:**

Poor sleepers showed uniformly higher PRO-PD burden, with a non-motor phenotype consisting of insomnia, fatigue, anxiety, and depression consistent with prior reports. The PRO-PD further identified additional equally-correlated symptoms, such as poor balance, lacking motivation, and social withdrawal providing expanded perspective. These findings highlight the need for routine sleep assessments in PD management and suggest that interventions targeting sleep disturbances may alleviate symptom burden and improve QoL. Future longitudinal studies could help establish causality and explore therapeutic strategies to improve sleep quality in PD.

## Introduction

Parkinson’s disease (PD), the second most prevalent neurodegenerative disorder, is traditionally classified as a movement disorder, defined by motor symptoms, such as bradykinesia, rigidity, and tremor (Bloem et al., 2021). However, non-motor symptoms, particularly sleep impairments, which are among the most common non-motor symptoms, are increasingly recognized for their significant impact on patients’ quality of life (QoL) (Chaudhuri et al., 2006). Sleep impairments refer to disruptions in normal sleep patterns, including decreased sleep quality, prolonged sleep latency, reduced sleep duration, and excessive daytime sleepiness. These impairments often overlap with diagnosable medical sleep disorders, restless legs syndrome (RLS), rapid eye movement sleep behavior disorder (RBD), obstructive sleep apnea (OSA), insomnia, and circadian rhythm disorders. Population-based studies suggest the majority of individuals with PD have signs of sleep disorders (Zhang et al., 2020a; Garcia-Borreguero et al., 2003; Zhang et al., 2020b), and comprehensive reviews on the complex relationship between sleep disorders and PD pathophysiology exist elsewhere (Minakawa, 2022; Duret and Nagoshi, 2025), RBD in particular.(Zhou et al., 2024; Bohnen and Hu, 2019; Maggi et al., 2023).

The relationship between PD and sleep impairments is highly complex and potentially bidirectional. Certain sleep impairments, such as RBD, are recognized as prodromal markers of PD, often occurring years before the onset of motor symptoms (Al-Qassabi et al., 2017; Lysen et al., 2019). Neurodegeneration during the early stages of PD predominantly affects brainstem regions, including the subcoeruleus and pontine tegmentum, which are critical for REM sleep regulation (Braak et al., 2003). As the disease progresses, pathology extends to midbrain structures like the substantia nigra and eventually to cortical regions, worsening both sleep disturbances and motor symptoms. Recently, poor sleep quality has also been shown to exacerbate both motor symptoms and non-motor symptoms in patients already diagnosed with PD (Yi et al., 2022; Tang et al., 2022; Koh et al., 2022). Conversely, advanced motor symptoms themselves worsen sleep disorders, creating a bidirectional feedback loop that worsens the overall disease burden (Lima, 2013). This relationship is further complicated by confounding factors such as age, medication side effects, and comorbid neuropsychiatric conditions like depression and anxiety (Zhang et al., 2020b; Rutten et al., 2017).

Despite the known associations, the impact and clinical manifestations of sleep impairment on the overall disease severity remains underexplored. Limited studies suggest that poor sleep quality, assessed using tools like the Pittsburgh Sleep Quality Index (PSQI), correlates with worse disease outcomes, including dyskinesia, cognitive decline, and depression (Yi et al., 2022; Tang et al., 2022; Koh et al., 2022). However, assessing individual disease outcomes alone may lack the power to fully capture the cumulative impact of these symptoms on disease progression, and thus a cumulative score of disease severity may provide better insights. The Patient-Reported Outcomes in PD (PRO-PD) scale was developed precisely to address these gaps (Mischley et al., 2017). Unlike traditional scales like the Hoehn & Yahr (HY) and Unified PD Rating Scale (UPDRS), which are limited by their focus on motor symptoms and responsiveness to dopaminergic treatments, the PRO-PD is a patient-centered, continuous measure of disease severity (Zhao et al., 2010; Movement Disorder Society Task Force on Rating Scales for Parkinson’s Disease, 2003). It evaluates both motor and non-motor symptoms, incorporates patient-reported outcomes, and demonstrates a strong correlation with established PD severity scales, including years since diagnosis, HY stage, UPDRS, PD Questionnaire-39 (PDQ-39), Timed-Up-and-Go (TUG), and PROMIS Global measures (Mischley et al., 2017). Furthermore, the PRO-PD’s cumulative scoring system allows for symptom stratification across different subcategories and provides a comprehensive assessment of disease progression. The validity of PRO-PD in tracking PD symptom severity was further reinforced by a recent longitudinal study conducted in a representative outpatient sample of 286 PD patients (von Below et al., 2023). The study demonstrated excellent internal consistency (Cronbach’s *α*: 0.93), good test–retest reliability (ICC: 0.87), and strong convergent validity with established PD measures (correlations: 0.69–0.71). Furthermore, a minimal clinically important change (MCIC) of 119 units was identified for the PRO-PD score, indicating the threshold at which symptom changes become clinically meaningful. This underscores its sensitivity to disease progression. By capturing patient-reported symptoms across a broad spectrum, PRO-PD provides a comprehensive view of disease burden and highlights symptom clusters that may represent sleep-related phenotypes.

The present analysis examines the relationship between overall sleep quality, assessed using PSQI scores, and disease severity, assessed using PRO-PD scores, in 498 patients with idiopathic PD (IPD). The analysis is based on baseline data from a prospective cohort study on patients with self-reported IPD. In addition to exploring the association between sleep quality and overall disease severity, the analysis also evaluates the relationship between specific sleep quality parameters and individual symptoms captured by the PRO-PD. This approach offers a more comprehensive understanding of how sleep disturbances are related to the disease burden in PD.

## Methods

### Study design and data collection

This study is a global cross-sectional survey of patients with self-reported IPD enrolled in 2023. The distribution of participants worldwide is detailed in Supplementary Table 1. It is part of a larger initiative utilizing data from the Modifiable Variables in Parkinsonism (MVP) study, which was initiated in 2013.^1^ The MVP is an ongoing, prospective, internet-based natural history study designed to identify modifiable factors associated with PD progression and symptom severity. To date, more than 3,500 individuals with parkinsonism have been enrolled (Mischley, 2024).

The study received approval from the Bastyr University Institutional Review Board (IRB #13A-1332) and is registered on ClinicalTrials.gov (NCT02194816). The IRB granted a Waiver of Documentation of Informed Consent, allowing participants to provide consent electronically after reviewing the Participant Information Sheet.

Data collection and management were conducted using Research Electronic Data Capture (REDCap), a secure, web-based application designed for building and managing online surveys and databases. The REDCap tools, hosted at Bastyr University, facilitated efficient data entry, validation, and storage while ensuring compliance with data security and confidentiality standard. The ongoing study collects data on more than 300 variables through a 60–90 min survey, including multiple-choice, open-ended, and scale-based formats. Participants complete the survey twice a year, with the flexibility to skip questions and complete it over multiple sittings. For this analysis, only the most recent survey responses were used.

### Study variables

For the present study, data were extracted for demographic and clinical variables to assess factors associated with PD severity. Demographic variables included age, gender, ethnicity/race, education, and income. Clinical variables comprised measures of disease severity, sleep quality, and years since PD diagnosis. The primary outcome of the study was disease severity, assessed using the PRO-PD score, whereas sleep quality, evaluated using the Pittsburgh Sleep Quality Index (PSQI), served as the main independent variable.

### Disease severity

Disease severity was assessed using the PRO-PD score, which was computed from data collected through the PRO-PD questionnaire. This questionnaire evaluates 33 common PD symptoms, including both motor and non-motor symptoms, based on the participant’s self-assessment of symptoms over the past week. Each symptom was rated on an unnumbered sliding scale ranging from 0 (optimal health) to 100 (debilitation). The total PRO-PD score is the cumulative sum of all symptom ratings, ranging from 0 to 3,300, with each symptom weighted equally. Notably, most of the symptoms exhibit a correlation of less than 0.4 (Spearman’s-*ρ*) among themselves, highlighting the importance of capturing a diverse range of PD symptoms (Supplementary Figure 1).

### Sleep quality

Sleep quality was assessed using the PSQI, a validated self-reported questionnaire designed to evaluate sleep quality and disturbances over the past month. The PSQI consists of 19 items generating seven component scores, including subjective sleep quality, sleep latency, sleep duration, habitual sleep efficiency, sleep disturbances, use of sleep medications, and daytime dysfunction. Each component is scored on a 0 to 3 scale, with a total PSQI score ranging from 0 to 21 (Mollayeva et al., 2016; Schrempf et al., 2014). Correlations among the seven PSQI components were found to be less than 0.5 (Spearman’s ρ) among all seven PSQI components within the study population (Supplementary Figure 2).

Higher PSQI scores indicate poorer sleep quality, with a cutoff of >5 commonly used to define clinically significant sleep impairment. A PSQI score between 5 and 10 suggests mild sleep difficulties, whereas a score of 11–21 is typically considered indicative of significant sleep problems requiring further clinical attention.

### Statistical analysis

Statistical analyses were conducted using R version 4.4.2 (“Pile of Leaves”). Only patients with complete data on the PSQI and PRO-PD scores were included in the analysis. The normality of continuous variables was assessed using the Shapiro–Wilk test. A *p*-value < 0.05 was considered statistically significant for all analyses.

In the univariable analysis, associations between the PSQI score and all independent variables were examined using appropriate statistical tests. For continuous independent variables, Pearson’s correlation was used if the data were normally distributed, and Spearman’s rank correlation was used otherwise. For categorical independent variables, the Mann–Whitney U test was applied for two-group comparisons, while the Kruskal-Wallis test was used for comparisons across more than two groups. Only variables that showed statistical significance in univariable analysis were carried forward to the multivariable linear regression analysis.

Multivariable linear regression was used to examine the relationship between PSQI score and the selected independent variables. Outliers were identified using Cook’s distance, and a sensitivity analysis was performed to evaluate their influence on the regression model.

As an exploratory analysis, associations between PRO-PD scores (both motor and non-motor symptoms) and individual PD symptoms with PSQI scores, including its components, were further evaluated. Pearson’s or Spearman’s correlation was used to assess relationships between individual PD symptoms and PSQI scores, while the Kruskal-Wallis test was used to examine differences in PRO-PD motor and non-motor symptoms across sleep quality categories. To account for multiple testing, False Discovery Rate (FDR) correction was applied using the Benjamini-Hochberg method to control for Type I errors.

## Results

A total of 703 participants enrolled in the study during 2023. Among them, 524 (75% of total enrolled) reported a diagnosis of IPD. Of these, 193 participants did not provide information on their sleep patterns and behaviors, making it impossible to compute their PSQI scores; they were subsequently excluded. The remaining 331 participants (47% of total enrolled) fully completed the PRO-PD questionnaire and were included in the analysis investigating the association between sleep quality and disease severity in the present study.

### Descriptive analysis

**Figure 1:**
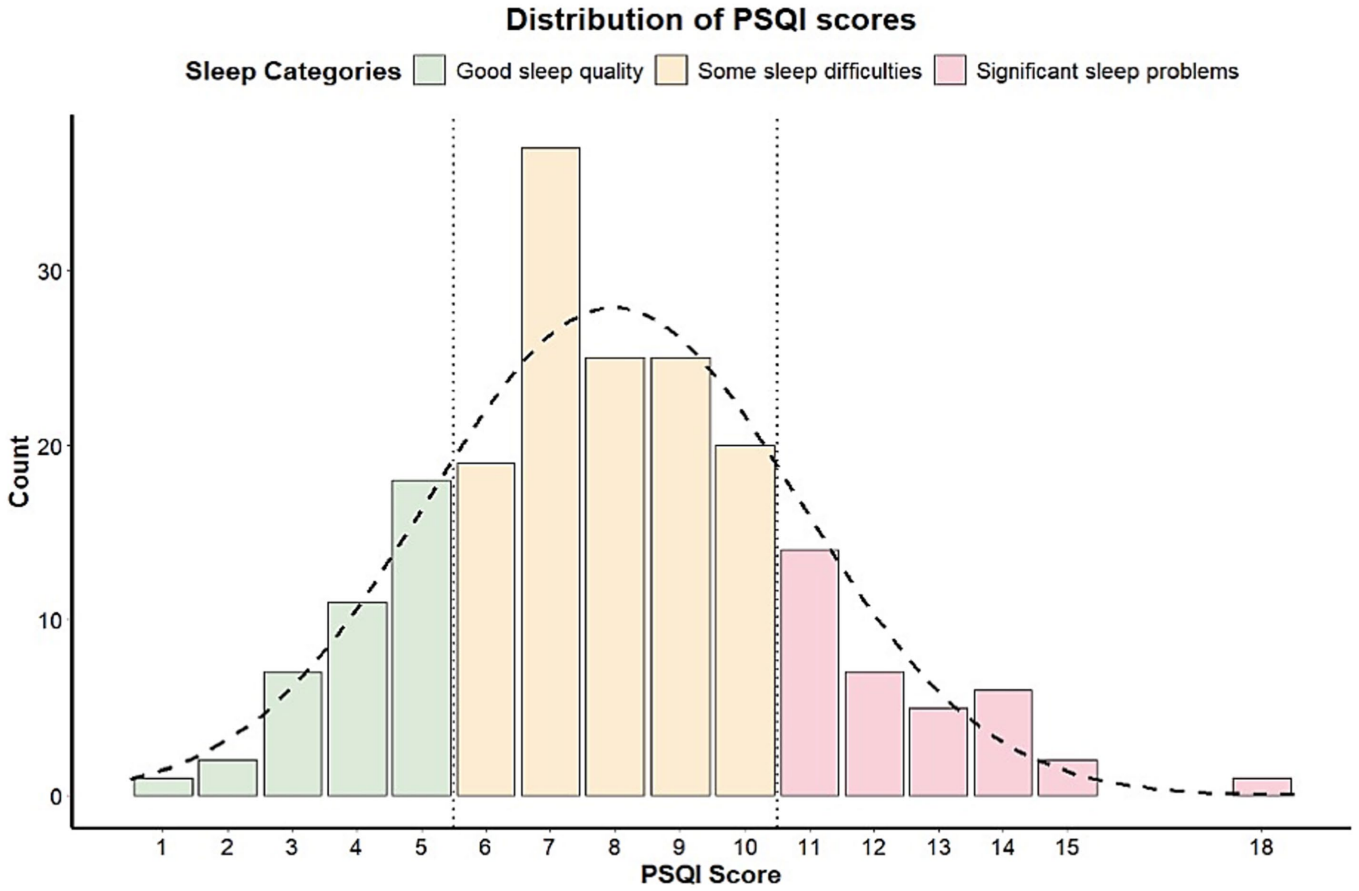
Distribution of PSQI scores among different sleep quality categories in idiopathic patients with PD.

The demographic and clinical characteristics of the study population have been provided in the Table 1. The participants were predominantly older adults, with a median age of 68.1 years, ranging from 39.3 to 89.9 years. Interestingly, contrary to typical PD population demographics, females were overrepresented in this cohort, comprising 59.5% of participants. Individuals participating in this online survey came from 22 countries, although 234 (70.6%) were from the United States (Supplementary Table 1). Participants had been living with a PD diagnosis for a median duration of 7.8 years, with durations ranging from less than a year to 36.1 years. A significant proportion of participants had achieved a graduate or professional degree (51.4%), and more than 60% reported an annual income exceeding $60,000 USD. Sleep quality, PSQI, demonstrated variability among participants. The median PSQI score was 7.0, with scores ranging from 1 to 18. Notably, only about one in three participants reported good sleep quality, as reflected in PSQI scores of 5 or below (Figure 1). Disease severity, measured using the PRO-PD scale, also displayed a wide range, with participants reporting a median score of 699, spanning from 50 to 2,290. These findings highlight the considerable variability in both sleep quality and disease severity across the study population. Correlation plots showing the relationship between PRO-PD components and PSQI domains can be found in Supplementary Figures 1, 2, respectively.

**Table 1:** Demographic and clinical characteristics of the study population.

Characteristics	Idiopathic PD (*N* = 331)
Age (years)	
Mean (SD)	67.5 (8.74)
Median [Min, Max]	68.1 [39.3, 89.9]
Not available	8 (2.4%)
Gender	
Male	132 (39.9%)
Female	197 (59.5%)
Not available	2 (0.6%)
Years since PD diagnosis	
Mean (SD)	8.44 (5.68)
Median [Min, Max]	7.80 [0.00548, 36.1]
Not available	12 (3.6%)
Ethnicity/Race	
White	308 (93.1%)
Non-White	20 (6.0%)
Not available	3 (0.9%)
Education	
Grades 9–11	2 (0.6%)
Completed High School/GED	21 (6.3%)
Technical school	17 (5.1%)
Associate degree	23 (6.9%)
Bachelor’s degree	97 (29.3%)
Graduate/Professional degree	170 (51.4%)
Not available	1 (0.3%)
Income	
Less than $20,000	9 (2.7%)
Between $20–40,000	29 (8.8%)
Between $40–60,000	38 (11.5%)
Between $60–80,000	47 (14.2%)
Between $80–100,000	37 (11.2%)
Between $100–150,000	70 (21.1%)
More than $150,000	56 (16.9%)
Not available	45 (13.6%)
PRO-PD	
Mean (SD)	793 (467)
Median [Min, Max]	699 [50.0, 2,290]
PSQI score (continuous)	
Mean (SD)	7.80 (3.66)
Median [Min, Max]	7.00 [1.00, 18.0]
Sleep quality (PSQI score range)	
Good sleep quality (0–5)	98 (29.6)
Some sleep difficulties (6–10)	154 (46.5)
Significant sleep problems (11–21)	79 (23.9)

### Univariable association analysis

**Figure 2:**
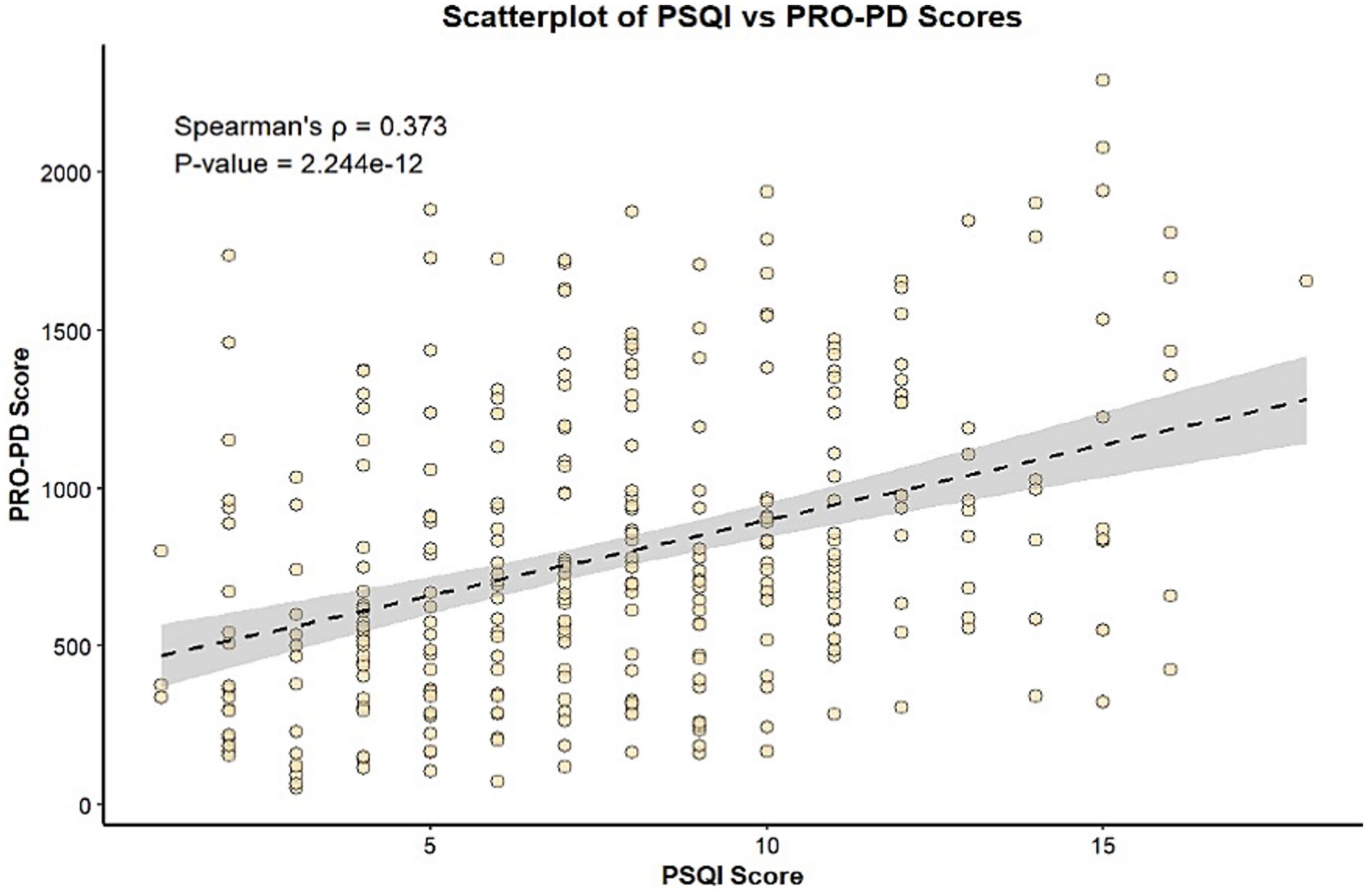
Scatter plot showing correlation between sleep quality and disease severity in in idiopathic patients with PD.

Despite the non-normal distribution of both PSQI and PRO-PD scores, as demonstrated by statistical tests (data not shown), a clear linear trend was observed in the scatterplot between the two variables. This relationship was further supported by a statistically significant positive correlation, as indicated by Spearman’s coefficient (rho = 0.373, *p* = 2.24 × 10^−12^) (Figure 2). A similarly strong positive correlation of PSQI was also observed with years since diagnosis, along with a slightly weaker but still significant positive correlation with age (Table 2).

**Table 2:** Univariable association analysis of PRO-PD with continuous dependent variables.

Dependent variable	*ρ* (Spearman’s rho)	*p*-value*
Age (*n* = 323)	0.199	3.13E-03
Years since diagnosis (*n* = 319)	0.317	6.75E-09
PSQI score (*n* = 331)	0.373	2.24E-12

*Computed using Spearman’s rank correlation test.

The effect of other covariates on PRO-PD demonstrated a nominally significant association with gender, where females had a considerably lower median PRO-PD score (665) compared to males (827.5) (Table 3). Additionally, income was associated with PRO-PD, with individuals earning a low to moderate income (20,000–60,000 USD per year) exhibiting higher PRO-PD scores, whereas those with very high income (>100,000 USD per year) had the lowest PRO-PD scores. This gap between low and high PRO-PD scores was particularly evident among individuals with significant sleep problems, who had a median score of 959 (range: 654.5 to 1365.0), compared to those with good sleep quality (median: 499, range: 295.3 to 805.5; *p* = 2.23 × 10^−10^).

**Table 3:** Univariable association analysis of PRO-PD with categorical dependent variables.

Dependent variable	PRO-PD		*p*-value*
	Mean (SD)	Median [Min, Max]	
Ethnicity/Race			
White (308)	783.6 (462.7)	698.5 [423.0, 1059.3]	0.814
Non-White (20)	795.0 (403.5)	639.7 [533.5, 1005.3]	
Gender (n)			
Male (197)	854.0 (477.5)	827.5 [505.8, 1194.0]	**0.0486**
Female (132)	757.2 (456.4)	665.0 [422.0, 986.0]	
Education (*n*)			
Grades 9–11 (2)	1880.0 (579.8)	1880.0 [1675.0, 2085.0]	0.179
Completed High School/GED (21)	979.2 (572.5)	1150.0 [471.2, 1358.0]	
Technical school (17)	781.2 (413.0)	684.0 [449.0, 960.0]	
Associate degree (23)	765.1 (420.6)	748.0 [547.5, 946.5]	
Bachelor’s degree (97)	754.7 (414.5)	711.0 [438.0, 992.0]	
Graduate/Professional degree (170)	778.8 (471.3)	671.5 [423.0, 993.8]	
Income (n)			
Less than $20,000 (9)	753.7 (560.0)	610.0 [326.0, 1150.0]	**0.0315**
Between $20–40,000 (29)	924.9 (471.1)	931.0 [570.0, 1150.0]	
Between $40–60,000 (38)	856.1 (410.7)	831.7 [545.3, 1237.3]	
Between $60–80,000 (47)	825.5 (407.0)	727.0 [519.5, 1096.0]	
Between $80–100,000 (37)	888.4 (511.8)	762.0 [533.0, 1375.0]	
Between $100–150,000 (70)	706.0 (479.0)	659.5 [295.3, 928.8]	
More than $150,000 (56)	654.4 (399.6)	627.5 [362.0, 834.5]	
Sleep quality (PSQI score range, *n*)			
Good sleep quality (0–5, 98)	587.4 (415.5)	499.0 [295.3, 805.5]	**2.23E-10**
Some sleep difficulties (6–10, 154)	798.6 (434.9)	726.5 [480.9, 992.8]	
Significant sleep problems (11–21, 79)	1038.8 (470.4)	959.0 [645.5, 1365.0]	

*Computed using the Mann–Whitney U test or Kruskal-Wallis test.

Bold values indicate *p*-value < 0.05.

### Multivariable regression analysis

To rule out the effect of confounding, the independent association of PRO-PD scores with PSQI was examined by adjusting for significant covariates identified in the univariable analysis, namely age, gender, income, and years since diagnosis, using multiple linear regression (Table 4). A similarly strong association between PSQI and PRO-PD was observed, where each unit increase in the PSQI score (indicating poorer sleep quality) was associated with an estimated 39.7-unit increase in the PRO-PD score. This finding was highly significant, suggesting that individuals with poor sleep quality experience significantly more severe symptoms of IPD (*p* = 1.25 × 10^−8^). While age was no longer significantly associated with PRO-PD, years since diagnosis remained a strong predictor, with each additional year leading to a 21.4-unit increase in PRO-PD scores. Gender differences were also evident, with males having PRO-PD scores that were 119.2 points higher than females, indicating more severe symptoms among men. Additionally, income also retained its association with PRO-PD. A sensitivity analysis was conducted by removing 20 influential data points from the multiple linear regression examining the association between PSQI and PRO-PD. This resulted in a much stronger association (*p* = 8.25 × 10^−11^).

**Table 4:** Multivariable linear regression analysis of PRO-PD with dependent variables.

c	Estimate	SE	*p*-value
PSQI	39.7	6.7	**1.25E-08**
Years since PD diagnosis	21.4	4.3	**1.18E-06**
Gender			
Male	Ref.		
Female	−119.2	49.5	**0.017**
Age (Years)	5.0	2.8	0.079
Income			
Income less than $20,000	Ref.		
Income between $20–40,000	257.5	92.1	**0.006**
Income between $40–60,000	79.3	84.9	0.351
Income between $60–80,000	122.4	78.0	0.118
Income between $80–100,000	188.2	81.7	**0.022**
Income less than $20,000	225.3	149.8	0.134
income more than $150,000	−33.2	72.9	0.649

Adjusted *R*-square (Model): 0.2418.

*p*-value (Model): 1.86E-13.

Bold values indicate *p*-value < 0.05.

**Figure 3:**
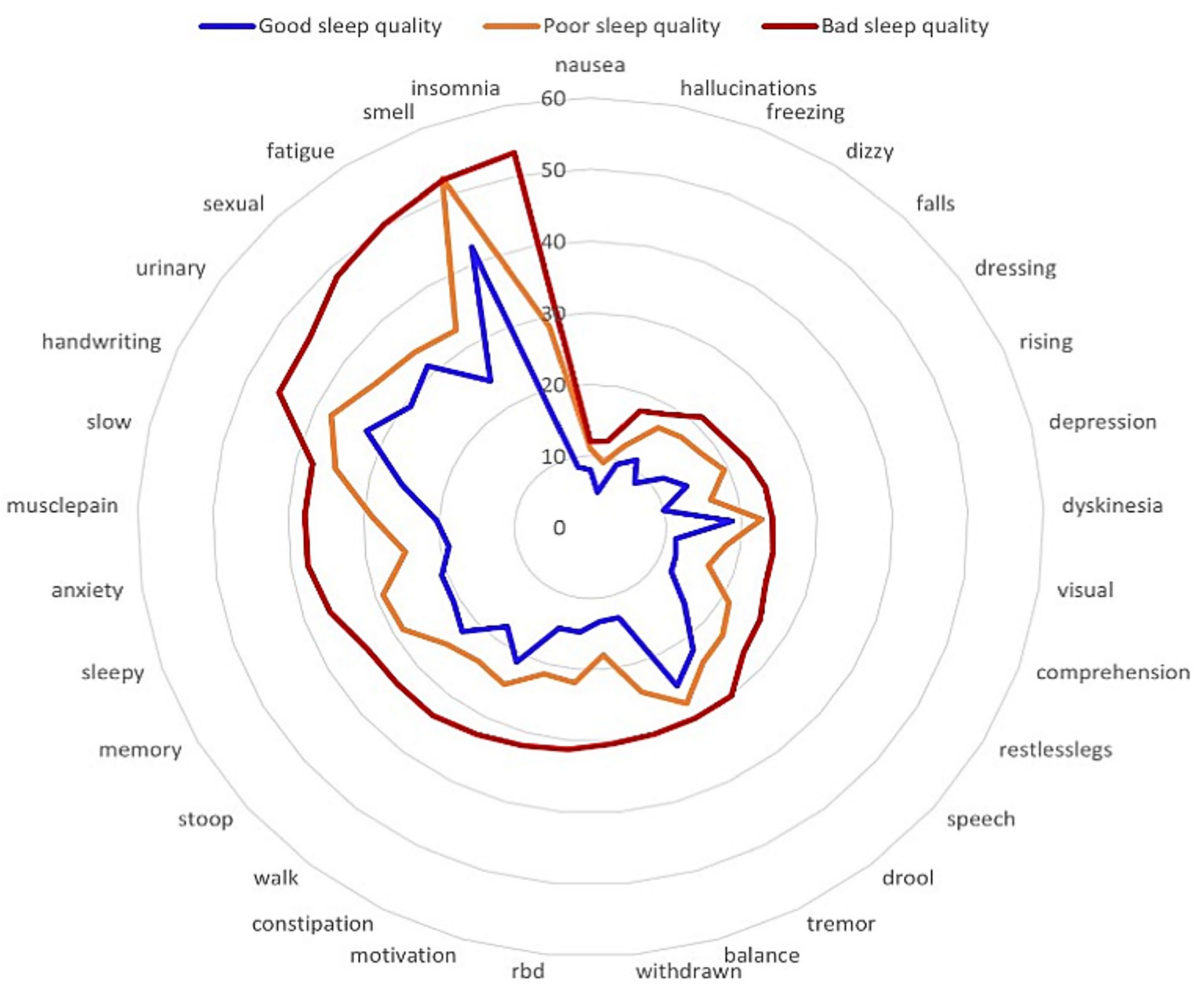
Radar plot showing disease symptoms associated with different sleep quality categories.

The association between overall PSQI scores and individual PD symptoms showed the strongest correlation with insomnia (*ρ* = 0.69), fatigue (*ρ* = 0.37), anxiety (*ρ* = 0.32), impaired balance (*ρ* = 0.30), apathy (*ρ* = 0.29), depression (*ρ* = 0.29), and myalgia (*ρ* = 0.28), whereas weak correlations were observed for tremor, hyposmia, drooling, and orthostatic hypotension (Spearman-ρ < 0.15) The radar plot lists all PRO-PD symptoms, in counter-clockwise order from greatest to least symptom severity, in bad sleepers. The plot provides visual demonstration that all PRO-PD symptoms are rated as more severe as sleep quality diminishes (Table 5; Figure 3). The association between overall PRO-PD score with individual PSQI components showed strongest significant correlation between PRO-PD and daytime dysfunction (*ρ* = 0.51), sleep disturbances (*ρ* = 0.29), and subjective sleep quality (*ρ* = 0.21). Use of sleeping medication, sleep duration, and sleep latency with significantly, but only modestly (*ρ* < 0.20) (Table 6). When PRO-PD components were compared to individual PSQI components, daytime dysfunction was the strongest and most consistent correlate, with non-motor symptoms fatigue, anxiety, depression, apathy, and myalgia all being associated across multiple PSQI domains (Supplementary Table 2). The myalgia variable, defined as “muscle cramping, pain, or aching: most common in the morning or as medications are wearing off,” was associated with PSQI components sleep disturbances, use of sleeping medications, habitual sleep efficiency, and daytime dysfunction, suggesting pain relief and preventing wearing off of overnight medication may be a path to improved sleep.

**Table 5:** Association of PRO-PD components with overall PSQI score.

PRO-PD component*	Spearman’s-*ρ*	*p*-value
Insomnia	0.69	<2.2E-16
Fatigue	0.37	3.62E-12
Anxiety	0.32	3.30E-09
Balance	0.30	1.78E-08
Withdrawn	0.29	5.14E-08
Motivation	0.29	5.16E-08
Depression	0.29	6.08E-08
Muscle pain	0.28	1.53E-07
Sleepy	0.28	2.40E-07
Walk	0.27	4.81E-07
Visual	0.24	1.40E-05
Slow	0.22	4.05E-05
Memory	0.22	4.09E-05
Rest less legs	0.22	5.69E-05
Dressing	0.22	7.70E-05
RBD	0.21	9.72E-05
Stoop	0.21	9.84E-05
Urinary	0.21	0.000127962
Comprehension	0.21	0.000161484
Speech	0.20	0.0001791
Rising	0.20	0.000273095
Nausea	0.19	0.000463887
Handwriting	0.19	0.000619677
Constipation	0.18	0.000729275
Falls	0.18	0.001021434
Sexual	0.17	0.001487732
Freezing	0.17	0.002119654
Dizzy	0.14	0.01221949
Hallucinations	0.13	0.016063317
Dyskinesia	0.13	0.022437369
Drool	0.11	0.037488109
Smell	0.10	0.064103656
Tremor	0.07	0.18510812

*Components sorted in the order of increasing *p*-values.

**Table 6:** Association of PSQI components with overall PRO-PD scores.

PSQI-component	Spearman-*ρ*	*p*-value
Daytime dysfunction	0.51	<2.2E-16
Sleep disturbances	0.29	9.87E-08
Subjective sleep quality	0.21	0.0002
Use of sleeping medications	0.18	0.0010
Sleep duration	0.17	0.0016
Habitual sleep efficiency	0.16	0.0032
Sleep latency	0.12	0.0301

## Discussion

As has been previously reported, there was a high prevalence of sleep impairments, with most participants reporting PSQI scores indicative of poor sleep quality. The findings further demonstrate a significant association between poor sleep quality and increased patient-reported symptom severity, a nuanced look at the poor sleep symptom phenotype, and suggest a three-point change in PSQI score could be considered clinically meaningful in PD. Specifically, the analysis revealed that a one-unit increase in PSQI was associated with a 39.7-unit increase in PRO-PD, suggesting that worsening sleep quality directly impacts overall PD severity or that disrupted sleep is a core feature of the syndrome. Given that a recent study determined the MCIC in PRO-PD to be 119 units, a three-unit increase in PSQI could independently result in a clinically meaningful change in PRO-PD scores. These results suggest that sleep disturbances are not merely a comorbid feature of PD but may actively contribute to disease progression, highlighting the need to address sleep disturbances in clinical practice.

Prior studies suggest that approximately half of people with PD have poor sleep, manifesting as longer wake up time after sleep onset, reduced sleep efficiency, more non-motor symptoms, and worse quality of life.(Jiang et al., 2023) Jiang et al. found 54.5% of drug-naïve individuals with PD were good sleepers, whereas only 29.6% of this cohort met those criteria, a discrepancy that may be explained by the difference in mean disease duration between the two studies (4 v. 8 years, respectively).(Jiang et al., 2023) Congruent and potentially related, a worsening of daytime fatigue was observed over a five-year observation period of drug-naïve patients.(Tholfsen et al., 2015).

The PSQI has been associated with overall symptom severity (HY: *r*^2^ = 0.166, *p* < 0.05) (Luo et al., 2023), as well as other sleep, quality of lilfe, and symptom severity scales, including Parkinson’s Disease Sleep Scale (PDSS), UPDRS-II (Activities of Daily Living), the Parkinson’s Disease Questionaire-39 (PDQ-39), and Non-Motor Symptoms Questionnaire (NMSQ).(Jiang et al., 2023; Boulamery et al., 2010) Prior studies have also reported worse motor symptoms,(Yi et al., 2022), and here we also report poor sleepers reported more problems with balance (*ρ* = 0.3), gait (*ρ* = 0.27), and slowness (*ρ* = 0.22). As the correlation between PSQI and PRO-PD score (Spearman’s *ρ* = 0.373, *p* < 0.001), is in the same direction and magnitude of previously reported associations between higher PSQI scores and HY stage (*r* = 0.223), UPDRS-I (*r* = 0.501, *p* < 0.01), UPDRS-III (*r* = 0.425, *p* < 0.01) (Qin et al., 2020), these results provide preliminary evidence of convergent validity for the PRO-PD as a measure of overall symptom severity in the context of sleep.

In terms of phenotype, prior studies have demonstrated statistically significant correlations between poor sleep and non-motor symptoms, specifically depression, anxiety, dysautonomia, and restless legs.(Yi et al., 2022; Koh et al., 2022; Rutten et al., 2017; Zhu et al., 2016; Amara et al., 2017) These results corroborate the association with fatigue, anxiety, and depression (*ρ* = 0.29–0.37), and correlation between PSQI and restless leg syndrome was *ρ* = 0.22 in this study, in line with a previously reported *ρ* = 0.254.(Qin et al., 2020) Symptoms associated with poor sleep identified by the PRO-PD, but not previously reported as part of a poor sleep phenotype include poor balance (*ρ* = 0.3), apathy (*ρ* = 0.29), muscle pain (*ρ* = 0.28) and visual disturbances (*ρ* = 0.24). This suggests the PRO-PD captures overlapping and additional aspects of disease burden beyond what has been captured by conventional severity scales.

There are many biologically plausible explanations by which sleep disturbances may be both a consequence and a risk factor for the onset and progression of PD. Degeneration of brainstem regions involved in sleep regulation contribute to disrupted sleep patterns (Braak et al., 2003). Animal models exhibit significant sleep fragmentation, reduced REM sleep, and altered circadian gene expression in the SCN and striatum (Boulamery et al., 2010; Hayashi et al., 2013; Lax et al., 2012; Kudo et al., 2011) and forced circadian disruption leads to aggravated motor deficits and increased neuronal loss (Liu et al., 2022; Majcin Dorcikova et al., 2023) Epidemiologically, sleep disturbances, such as RBD, insomnia, and excessive daytime sleepiness, frequently precede motor symptoms, suggesting that sleep dysfunction may serve as an early biomarker of disease onset (Lysen et al., 2019; Abbott et al., 2005; Postuma et al., 2019). These findings support a bidirectional relationship between sleep dysfunction and neurodegeneration, where sleep disturbances accelerate brain pathology, and neurodegeneration, in turn, exacerbates sleep dysfunction, forming a detrimental feedback loop.

Several other factors could further confound the relationship between sleep and disease severity in PD. For instance, motor symptoms, along with associated non-motor dysfunctions such as myalgia, nocturia, nightmares, and restless leg syndrome, could further disrupt sleep (Rijsman et al., 2014). Additionally, sleep problems can be worsened by discomfort resulting from nighttime hypokinesia, particularly difficulty turning in bed, leading to frequent awakenings and fragmented sleep (Stack and Ashburn, 2006). Meanwhile, pain and sensory disturbances associated with PD further exacerbate sleep disruption (Martinez-Martin et al., 2019). In addition, PD medications play a crucial role in managing motor symptoms, as dopaminergic therapies are essential for alleviating motor impairments may be associated with sleep disturbances (Nausieda et al., 1982; Paus et al., 2003). These medications have been linked to insomnia, fragmented sleep, and EDS. Higher dosages of dopamine agonists, in particular, increase the risk of daytime somnolence and sudden sleep episodes (O’Suilleabhain and Dewey, 2002). Additionally, while some PD medications can disrupt sleep, others may induce daytime drowsiness, further complicating the management of sleep-related symptoms in PD patients. Therefore, optimizing PD treatment requires a careful balance to effectively control motor symptoms while minimizing adverse effects on sleep.

This study has several strengths, including the use of validated instruments (PSQI and PRO-PD), a large sample size representative of the global population and comprehensive statistical analyses. However, limitations must be acknowledged. The PRO-PD was developed in English for the current study in 2013, (Mischley et al., 2017) and was translated to Swedish in 2014 and validated using the Swedish version in a West Swedish longitudinal cohort.(von Below et al., 2023) It is not known whether the scale translation, population reference ranges, or progression rates observed in Sweden are applicable globally. To enhance consistency, reliability, and accessibility of PRO-PD to researchers and clinicians, the non-profit organization Mapi Research Trust (Lyon, France) will oversee subsequent translation and validation efforts.

The cross-sectional design prevents causal inferences, and self-reported data may introduce recall bias. Additionally, the study cohort, derived from an online survey, may not fully represent the broader PD population, particularly those with limited digital literacy or access to healthcare. Future research should employ longitudinal study designs to establish causality between sleep disturbances and PD progression, and evaluate whether there are differences between early-onset and typical-onset PD (McCarter et al., 2023). Objective sleep measures, such as polysomnography, should be integrated into PD research to validate self-reported findings. Additionally, clinical trials examining the impact of sleep-targeted interventions on PD outcomes are warranted to inform evidence-based management strategies. Nevertheless, this study demonstrates a strong association between poor sleep quality and increased disease severity in PD patients. Integrating PRO-PD and sleep assessments into routine PD care may enable clinicians to better mitigate the broader impacts of PD symptoms and enhance overall patient well-being.

## Data Availability

The raw data supporting the conclusions of this article will be made available by the authors, without undue reservation.

## Footnotes

^1^ https://mvp-study.com/

## References

[ref1] Abbott R. D. Ross G. W. White L. R. Tanner C. M. Masaki K. H. Nelson J. S.. (2005). Excessive daytime sleepiness and subsequent development of Parkinson disease. Neurology 65, 1442–1446. doi: 10.1212/01.wnl.0000183056.89590.0d, PMID: 16275833

[ref2] Al-Qassabi A. Fereshtehnejad S. M. Postuma R. B. (2017). Sleep disturbances in the prodromal stage of Parkinson disease. Curr. Treat. Options Neurol. 19:22. doi: 10.1007/s11940-017-0458-1, PMID: 28466429

[ref3] Amara A. W. Chahine L. M. Caspell-Garcia C. Long J. D. Coffey C. Högl B.. (2017). Longitudinal assessment of excessive daytime sleepiness in early Parkinson’s disease. J. Neurol. Neurosurg. Psychiatry 88, 653–662. doi: 10.1136/jnnp-2016-315023, PMID: 28554959 PMC7282477

[ref4] Bloem B. R. Okun M. S. Klein C. (2021). Parkinson’s disease. Lancet 397, 2284–2303. doi: 10.1016/S0140-6736(21)00218-X 33848468

[ref5] Bohnen N. I. Hu M. T. M. (2019). Sleep disturbance as potential risk and progression factor for Parkinson’s disease. J. Parkinsons Dis. 9, 603–614. doi: 10.3233/JPD-191627, PMID: 31227656 PMC6700634

[ref6] Boulamery A. Simon N. Vidal J. Bruguerolle B. (2010). Effects of L-Dopa on circadian rhythms of 6-OHDA striatal lesioned rats: a radiotelemetric study. Chronobiol. Int. 27, 251–264. doi: 10.3109/07420521003664213, PMID: 20370468

[ref7] Braak H. Tredici K. D. Rüb U. de Vos R. A. I. Jansen Steur E. N. H. Braak E. (2003). Staging of brain pathology related to sporadic Parkinson’s disease. Neurobiol. Aging 24, 197–211. doi: 10.1016/S0197-4580(02)00065-9, PMID: 12498954

[ref8] Chaudhuri K. R. Healy D. G. Schapira A. H. (2006). Non-motor symptoms of Parkinson’s disease: diagnosis and management. Lancet Neurol. 5, 235–245. doi: 10.1016/S1474-4422(06)70373-8, PMID: 16488379

[ref9] Duret L. C. Nagoshi E. (2025). The intertwined relationship between circadian dysfunction and Parkinson’s disease. Trends Neurosci. 48, 62–76. doi: 10.1016/j.tins.2024.10.006, PMID: 39578132

[ref10] Garcia-Borreguero D. Larrosa O. Bravo M. (2003). Parkinson’s disease and sleep. Sleep Med. Rev. 7, 115–129. doi: 10.1053/smrv.2002.0229 12628213

[ref11] Hayashi A. Matsunaga N. Okazaki H. Kakimoto K. Kimura Y. Azuma H.. (2013). A disruption mechanism of the molecular clock in a MPTP mouse model of Parkinson’s disease. NeuroMolecular Med. 15, 238–251. doi: 10.1007/s12017-012-8214-x 23292542

[ref12] Jiang Y. Chen Y. Li D. Zhu S. Gu R. Wang Y.. (2023). Sleep structure and related clinical characteristics in drug-naive Parkinson’s disease with subjectively different sleep quality. Front. Neurol. 14:1156910. doi: 10.3389/fneur.2023.1156910, PMID: 37325221 PMC10264636

[ref13] Koh M. R. E. Chua C. Y. Ng S. Y. E. Chia N. S. Y. Saffari S. E. Chen R. Y. Y.. (2022). Poor sleep quality is associated with fatigue and depression in early Parkinson’s disease: a longitudinal study in the PALS cohort. Front. Neurol. 13:998103. doi: 10.3389/fneur.2022.998103, PMID: 36119701 PMC9476542

[ref14] Kudo T. Loh D. H. Truong D. Wu Y. Colwell C. S. (2011). Circadian dysfunction in a mouse model of Parkinson’s disease. Exp. Neurol. 232, 66–75. doi: 10.1016/j.expneurol.2011.08.003, PMID: 21864527

[ref15] Lax P. Esquiva G. Esteve-Rudd J. Otalora B. B. Madrid J. A. Cuenca N. (2012). Circadian dysfunction in a rotenone-induced parkinsonian rodent model. Chronobiol. Int. 29, 147–156. doi: 10.3109/07420528.2011.649870, PMID: 22324553

[ref16] Lima M. M. (2013). Sleep disturbances in Parkinson’s disease: the contribution of dopamine in REM sleep regulation. Sleep Med. Rev. 17, 367–375. doi: 10.1016/j.smrv.2012.10.006, PMID: 23481545

[ref17] Liu X. Yu H. Wang Y. Li S. Cheng C. al-Nusaif M.. (2022). Altered motor performance, sleep EEG, and Parkinson’s disease pathology induced by chronic sleep deprivation in Lrrk2(G2019S) mice. Neurosci. Bull. 38, 1170–1182. doi: 10.1007/s12264-022-00881-2, PMID: 35612787 PMC9554065

[ref18] Luo Y. Liu J. Chen D. Liu M. Yuan Y. Hu J.. (2023). How sleep quality affects activities of daily living in Parkinson’s disease: the mediating role of disease severity and the moderating role of cognition. Front. Aging Neurosci. 15:1238588. doi: 10.3389/fnagi.2023.1238588 37842121 PMC10570447

[ref19] Lysen T. S. Darweesh S. K. L. Ikram M. K. Luik A. I. Ikram M. A. (2019). Sleep and risk of parkinsonism and Parkinson’s disease: a population-based study. Brain 142, 2013–2022. doi: 10.1093/brain/awz113, PMID: 31038176 PMC6911221

[ref20] Maggi G. Vitale C. Cerciello F. Santangelo G. (2023). Sleep and wakefulness disturbances in Parkinson’s disease: a meta-analysis on prevalence and clinical aspects of REM sleep behavior disorder, excessive daytime sleepiness and insomnia. Sleep Med. Rev. 68:101759. doi: 10.1016/j.smrv.2023.101759, PMID: 36708642

[ref21] Majcin Dorcikova M. Duret L. C. Pottié E. Nagoshi E. (2023). Circadian clock disruption promotes the degeneration of dopaminergic neurons in male Drosophila. Nat. Commun. 14:5908. doi: 10.1038/s41467-023-41540-y, PMID: 37737209 PMC10516932

[ref22] Martinez-Martin P. Rizos A. M. Wetmore J. B. Antonini A. Odin P. Pal S.. (2019). Relationship of nocturnal sleep dysfunction and pain subtypes in Parkinson’s disease. Mov. Disord. Clin. Pract. 6, 57–64. doi: 10.1002/mdc3.12694, PMID: 30746417 PMC6335509

[ref23] McCarter S. J. Camerucci E. Mullan A. F. Stang C. D. Turcano P. St. Louis E. K.. (2023). Sleep disorders in early-onset parkinsonism: a population-based study. J. Parkinsons Dis. 13, 1175–1183. doi: 10.3233/JPD-230045, PMID: 37742659 PMC10657686

[ref24] Minakawa E. N. (2022). Bidirectional relationship between sleep disturbances and Parkinson’s disease. Front. Neurol. 13:927994. doi: 10.3389/fneur.2022.927994, PMID: 35923835 PMC9342689

[ref25] Mischley L. K. (2024). Modifiable variables in parkinsonism (MVP) study: Summary of study findings 2013–2023, 78.

[ref26] Mischley L. K. Lau R. C. Weiss N. S. (2017). Use of a self-rating scale of the nature and severity of symptoms in Parkinson’s disease (PRO-PD): correlation with quality of life and existing scales of disease severity. NPJ Parkinsons Dis. 3:20. doi: 10.1038/s41531-017-0021-5, PMID: 28649620 PMC5473828

[ref27] Mollayeva T. Thurairajah P. Burton K. Mollayeva S. Shapiro C. M. Colantonio A. (2016). The Pittsburgh sleep quality index as a screening tool for sleep dysfunction in clinical and non-clinical samples: a systematic review and meta-analysis. Sleep Med. Rev. 25, 52–73. doi: 10.1016/j.smrv.2015.01.009, PMID: 26163057

[ref28] Movement Disorder Society Task Force on Rating Scales for Parkinson’s Disease (2003). The unified Parkinson’s disease rating scale (UPDRS): status and recommendations. Mov. Disord. 18, 738–750. doi: 10.1002/mds.10473, PMID: 12815652

[ref29] Nausieda P. A. Weiner W. J. Kaplan L. R. Weber S. Klawans H. L. (1982). Sleep disruption in the course of chronic levodopa therapy: an early feature of the levodopa psychosis. Clin. Neuropharmacol. 5, 183–194. doi: 10.1097/00002826-198205020-00003, PMID: 7139632

[ref30] O’Suilleabhain P. E. Dewey R. B. Jr. (2002). Contributions of dopaminergic drugs and disease severity to daytime sleepiness in Parkinson disease. Arch. Neurol. 59, 986–989. doi: 10.1001/archneur.59.6.986, PMID: 12056935

[ref31] Paus S. Brecht H. M. Köster J. Seeger G. Klockgether T. Wüllner U. (2003). Sleep attacks, daytime sleepiness, and dopamine agonists in Parkinson’s disease. Mov. Disord. 18, 659–667. doi: 10.1002/mds.10417, PMID: 12784269

[ref32] Postuma R. B. Iranzo A. Hu M. Högl B. Boeve B. F. Manni R.. (2019). Risk and predictors of dementia and parkinsonism in idiopathic REM sleep behaviour disorder: a multicentre study. Brain 142, 744–759. doi: 10.1093/brain/awz030, PMID: 30789229 PMC6391615

[ref33] Qin X. Li X. Chen G. Chen X. Shi M. Liu X. K.. (2020). Clinical features and correlates of poor nighttime sleepiness in patients with Parkinson’s disease. Parkinsons Dis. 2020:6378673. doi: 10.1155/2020/6378673 33005317 PMC7509546

[ref34] Rijsman R. M. Schoolderman L. F. Rundervoort R. S. Louter M. (2014). Restless legs syndrome in Parkinson’s disease. Parkinsonism Relat. Disord. 20, S5–S9. doi: 10.1016/S1353-8020(13)70004-X, PMID: 24262188

[ref35] Rutten S. Vriend C. van der Werf Y. D. Berendse H. W. Weintraub D. van den Heuvel O. A. (2017). The bidirectional longitudinal relationship between insomnia, depression and anxiety in patients with early-stage, medication-naive Parkinson’s disease. Parkinsonism Relat. Disord. 39, 31–36. doi: 10.1016/j.parkreldis.2017.01.015, PMID: 28365203 PMC5441947

[ref36] Schrempf W. Brandt M. D. Storch A. Reichmann H. (2014). Sleep disorders in Parkinson’s disease. J. Parkinsons Dis. 4, 211–221. doi: 10.3233/JPD-130301, PMID: 24796235

[ref37] Stack E. L. Ashburn A. M. (2006). Impaired bed mobility and disordered sleep in Parkinson’s disease. Mov. Disord. 21, 1340–1342. doi: 10.1002/mds.20944, PMID: 16773640

[ref38] Tang X. Yang J. Zhu Y. Gong H. Sun H. Chen F.. (2022). High PSQI score is associated with the development of dyskinesia in Parkinson’s disease. NPJ Parkinsons Dis. 8:124. doi: 10.1038/s41531-022-00391-y, PMID: 36175559 PMC9522669

[ref39] Tholfsen L. K. Larsen J. P. Schulz J. Tysnes O. B. Gjerstad M. D. (2015). Development of excessive daytime sleepiness in early Parkinson disease. Neurology 85, 162–168. doi: 10.1212/WNL.0000000000001737, PMID: 26085603

[ref40] von Below D. Wallerstedt S. M. Bergquist F. (2023). Validation of the Swedish patient-reported outcomes in Parkinson’s disease scale in outpatients. Mov. Disord. 38, 1668–1678. doi: 10.1002/mds.29517, PMID: 37382300

[ref41] Yi Q. Yu-Peng C. Jiang-Ting L. Jing-Yi L. Qi-Xiong Q. Dan-Lei W.. (2022). Worse sleep quality aggravates the motor and non-motor symptoms in Parkinson’s disease. Front. Aging Neurosci. 14:887094. doi: 10.3389/fnagi.2022.887094, PMID: 35754956 PMC9226540

[ref42] Zhang Y. Ren R. Sanford L. D. Yang L. Zhou J. Tan L.. (2020b). Sleep in Parkinson’s disease: a systematic review and meta-analysis of polysomnographic findings. Sleep Med. Rev. 51:101281. doi: 10.1016/j.smrv.2020.101281, PMID: 32135452

[ref43] Zhang Y. Zhao J. Huang D. Chen W. Yuan C. Jin L.. (2020a). Multiple comorbid sleep disorders adversely affect quality of life in Parkinson’s disease patients. NPJ Parkinsons Dis. 6:25. doi: 10.1038/s41531-020-00126-x, PMID: 33015354 PMC7492275

[ref44] Zhao Y. J. Wee H. L. Chan Y. H. Seah S. H. Au W. L. Lau P. N.. (2010). Progression of Parkinson’s disease as evaluated by Hoehn and Yahr stage transition times. Mov. Disord. 25, 710–716. doi: 10.1002/mds.22875, PMID: 20213822

[ref45] Zhou Y. Liu X. Xu B. (2024). Research Progress on the relationship between Parkinson’s disease and REM sleep behavior disorder. J. Integr. Neurosci. 23:166. doi: 10.31083/j.jin2309166, PMID: 39344226

[ref46] Zhu K. van Hilten J. J. Marinus J. (2016). The course of insomnia in Parkinson’s disease. Parkinsonism Relat. Disord. 33, 51–57. doi: 10.1016/j.parkreldis.2016.09.010, PMID: 27639814

